# Long-Term Contaminant Exposure Alters Functional Potential and Species Composition of Soil Bacterial Communities in Gulf Coast Prairies

**DOI:** 10.3390/microorganisms12071460

**Published:** 2024-07-18

**Authors:** Candice Y. Lumibao, Yue Liu

**Affiliations:** Department of Life Sciences, Texas A&M University—Corpus Christi, Corpus Christi, TX 78412, USA; yliu11@islander.tamucc.edu

**Keywords:** heavy metal pollution, oil pollution, South Texas, Gulf of Mexico, barrier islands, coastal dunes, bacterial diversity

## Abstract

Environmental pollution is a persistent threat to coastal ecosystems worldwide, adversely affecting soil microbiota. Soil microbial communities perform critical functions in many coastal processes, yet they are increasingly subject to oil and heavy metal pollution. Here, we assessed how small-scale contamination by oil and heavy metal impacts the diversity and functional potential of native soil bacterial communities in the gulf coast prairie dunes of a barrier island in South Texas along the northern Gulf of Mexico. We analyzed the bacterial community structure and their predicted functional profiles according to contaminant history and examined linkages between species diversity and functional potential. Overall, contaminants altered bacterial community compositions without affecting richness, leading to strongly distinct bacterial communities that were accompanied by shifts in functional potential, i.e., changes in predicted metabolic pathways across oiled, metal, and uncontaminated environments. We also observed that exposure to different contaminants can either lead to strengthened or decoupled linkages between species diversity and functional potential. Taken together, these findings indicate that bacterial communities might recover their diversity levels after contaminant exposure, but with consequent shifts in community composition and function. Furthermore, the trajectory of bacterial communities can depend on the nature or type of disturbance.

## 1. Introduction

Coastal environmental pollution is a persisting threat worldwide [1], adversely affecting both the abiotic environment as well as the biota of these habitats such as the coastal prairie dunes of barrier islands [1,2]. Barrier islands along the northern Gulf of Mexico, which border coastal shorelines separating oceans from inshore bays and estuaries, are projected to face increasing pressures from anthropogenic-driven stressors, from sea-level rise to pollution [3]. However, the impacts of chronic but systemic contaminations on its biotic communities have been less studied. As these islands provide an array of ecosystem services including protective barriers for the mainland against oceanic waves and structural framework for many coastal and estuarine habitats [4], they are among the most valuable yet vulnerable ecosystems worldwide [4,5]. Thus, understanding the consequences of environmental pollution on its constitutive biota such as soil microbes is critical for better management and restoration of these habitats. 

Soil microbial communities are highly diverse, playing major roles in coastal biogeochemical processes like soil carbon and nitrogen cycling. They also serve as bioindicators for soil health [6]. Due to their high diversity, it has long been assumed that soil microbial communities exhibit functional redundancy, i.e., different co-existing microbial taxa perform similar functions [7], such that changes in community composition do not alter their functional potential or diversity. Recent studies suggest, however, that species composition and functional potential are not always linearly associated [8,9] as some metabolic functions appear to be decoupled from species assemblages, i.e., high microbial diversity support different functions with limited redundancy [10]. Such decoupling of microbial functional potential (herein, functional diversity) from species composition raises critical questions regarding the relative importance of species diversity vs. functional diversity in understanding community responses to the environmental changes. It is thus necessary to investigate linkages between microbial species composition and function subject to environmental disturbances like pollution for predicting community responses and resilience. 

Oil and heavy metal contaminations can alter both the composition and function of soil microbial communities immediately, and over time. They can impact the community structure, for example, by promoting growth of chemical-tolerant microbes, thereby reducing the overall species diversity following exposure [11], but without the loss of functional diversity potential [12]. It is, however, possible to have altered functional potential of microbial communities if highly specialized microbial groups are selected [12,13], especially after long-term exposure to contaminants. For instance, different concentrations of heavy metals like lead (Pb) and zinc (Zn) can create microbial communities comprised by microbes with different degradation capabilities [14,15]. These resistant bacteria can easily adapt and increase in abundance, altering microbial community structure [15]. Moreover, increases in certain bacterial phyla that harbor large suites of metal-resistance genes such as *Pseudomonadota*, *Bacteroidota*, and *Bacillota* (formerly, Firmicutes) but not in other phyla will lead to shifts in indigenous soil microbial communities and, thus, overall functions of the community [16]. 

However, different types of contaminants like heavy metal and petroleum or oil (including polycyclic aromatic hydrocarbons (PAHs)) can have dissimilar magnitude of effects on soil microbiota. For instance, some studies have shown that petroleum has higher negative impacts on bacterial functions and activities [17] such as dehydrogenase activity without altering diversity than metal contaminants [18,19]. Within oil-contaminated environments, hydrocarbon type and exposure time have been shown to determine the microbial response to pollution. For example, *Oceanospirillales* and *Pseudomonadales* show increased dominance in the presence of high aliphatic content of the pollution, whereas *Alteromonadales*, *Flavobacteriales*, and *Rhodobacterales* dominate polyaromatic polluted samples [20]. It is, however, often challenging to have a side-by-side comparison of soil microbial response to different contaminant types, i.e., oil and heavy metal as most pollutions span large spatial areas, where soil heterogeneity can muddle patterns, and/or these contaminants are mixed, hence resulting in the additive effects of interacting contaminants [19]. 

We investigated how exposure to small-scale but chronic contaminants, i.e., heavy metal and oil pollution, affect the soil bacterial communities in the gulf coast prairie marsh and dune habitats of a barrier island in South Texas, USA. We focused on sites that are in close proximity but with separate known history of small-scale contaminations from previous oil or industrial activities. We tested the following two hypotheses:

**H1:**  *Long-term contaminant exposure reduces the species diversity and alters the functional potential of soil bacterial communities, with more specialized bacteria found in contaminated soils* *; and*

**H2:**  *The magnitude of shifts in the diversity, composition, and functional potential of soil microbial communities will differ according to contaminant history.*

Addressing these hypotheses will provide insights into the impacts of long-term exposure to contaminants on soil bacterial communities. 

## 2. Materials and Methods

### 2.1. Site and Soil Collection

The site is in the gulf prairie dune and marsh habitats of a barrier island, Mustang Island State Park in South Texas, USA along the Gulf of Mexico. Soil samples were collected from three sites or areas located the back part of the Mustang Island: (1) a former oil well pad with localized oil leakage since 2016 (hereafter, oiled environment); (2) a nearby area with known heavy metal contamination since 2013, i.e., with significant levels of barium (Ba) and lead (Pb) in the soil (hereafter, metal environment) resulting from previous activities (2013 Hanson Report and 2018 Texas Parks and Wildlife Department Prism Report, pers comm.); and (3) another nearby undisturbed and uncontaminated area (hereafter, control or uncontaminated). All sites were located on the back dune of the Mustang Island, with each site encompassing ~1 km^2^ area. Previous sediment testing in 2018 of the M2 site indicated barium (Ba), arsenic (As), lead (Pb), and other heavy metals present in the area (2013 Hanson Report and 2018 Texas Parks and Wildlife Department Prism Report). 

From each site, ~15 soil cores across three random transects were collected from the top 6–12 cm soil (total = 45). Latitude and longitude coordinates were recorded for each soil sample (Table S1). Soil samples were stored in 50 mL tubes and immediately transferred to a cooler while in the field. Samples were then stored at −20 °C in the laboratory prior to DNA extraction. The remaining soil samples after DNA extraction were air-dried and sent to Louisiana State University (LSU) Agriculture Center for analyses of soil nutrient content (total soil carbon (%C) and nitrogen (%N), potassium (K, mg/kg), zinc (Zn, mg/kg), copper (Cu, mg/kg), calcium (Ca, mg/kg), magnesium (Mg, mg/kg), phosphorous (P, mg/kg), sodium (Na, mg/kg), and sulfur (S, mg/kg)) and pH (Table S2). 

### 2.2. Bacterial Community Assessment

Soil bacterial communities were profiled by extracting, amplifying, and next-generation sequencing of the V5–V6 region of 16S rRNA. Microbial genomic DNA was extracted from soil samples using Qiagen DNeasy PowerSoil Pro kit (Hilden, Germany) following the manufacturer’s protocol with slight modification. Briefly, 25 milligrams (mg) of soil samples were subjected to bead beating and incubated at 65 °C, then extraction was carried out as specified in the manufacturer’s protocol. DNA libraries were created using a two-step PCR protocol described elsewhere [21,22,23]. We normalize the amount of DNA across all samples to 10 nanograms prior to the first PCR. The first PCR amplifies the V5-V6 16S rRNA region using the modified Illumina adapter and gene primers 799F (5′ CACTCTTTCCCTACACGACGCTCTTCCGATCTAACMGGATTAGATACCCKG 3′) and 1115R (5′ GTGACTGGAGTTCAGACGTGTGCTCTTCCGATCTAGGGTTGCGCTCGTTG 3′), with the following conditions: initial denaturation 95 °C 5 min, 30 cycles of 98 °C 20 s, 52–56 °C 15 s and 72 °C for 30 s; final elongation at 52 °C for 5 min. For each sample, PCR was performed in triplicate reactions at three different annealing temperatures (52, 54, and 56 °C) to remove amplification bias of certain taxa. Triplicate reactions for each sample were combined, and one µL of amplified products was subsequently dually indexed with unique eight base pair barcodes. Indexing PCR were carried out as follows: 9 °C initial denaturation, 9 cycles of 98 °C for 15 s, 50 °C for five seconds, and 72 °C for 20 s and final elongation at 72 °C for one minute. Indexed libraries were purified using Qiaquick PCR Purification Kit (Qiagen, Hilden, Germany) and quantified using the Quant-iT^®^ dsDNA HS Assay kit with a Qubit Flourometer (ThermoFisher Scientific, Waltham, MA, USA). Samples were pooled in equimolar concentration (20 ng) and sequenced in the Illumina MiSeq 2 × 300 paired end sequencing platform at Texas A&M University AgriLife. Two negative controls were included in the sequencing. 

MiSeq sequence analysis was conducted using QIIME2 [24]. Prior to denoising, paired sequences were filtered for quality and adaptors/distal priming sites were trimmed and removed using CUTADAPT v3.4 [25]. Denoising was performed using the DADA2, which implements joining, quality filtering, and chimera detection. Data were denoised with reverse reads truncated at 220 and forward at 160 and with error rates of 0.23. Chimeras were removed and the sequence table was generated. The resulting reads were clustered into Amplicon Sequences Variants (ASVs) using the DADA2 pipeline [26] implemented in Qiime2. The taxonomy of ASVs was assigned against the reference SILVA 132 ribosomal RNA gene database [27] using the QIIME2 feature classifier plugin classify-sklearn naïve Bayes classifier trained on the V5-V6 gene region. As some low-abundance ASVs could be a product of amplification and/or sequencing artefacts, ASVs with total abundances <4 across all samples were removed from downstream analyses. We predicted metabolic potential of bacterial communities (i.e., functional potential) based on abundances of MetaCyc metabolic pathways associated with each community using PICRUSt2 v2.5.2 [28]. Prior to downstream analyses, the metabolic data were center-log transformed using the clr function in compositions package [29] in R v.4.2.3.

### 2.3. Bacterial ASV Diversity and Composition Analyses

Bacterial ASV diversity (i.e., alpha diversity; hereafter, species diversity) was calculated using effective number of species (*ENS_PIE_*). For comparison, we also calculated Shannon diversity index. To test for whether contaminant type/history significantly altered soil bacterial communities, generalized linear model (glm) analyses were performed with *ENS_PIE_* diversity as response variable and contaminant type (metal, oiled, or uncontaminated or control) as fixed variables. As soil properties can influence microbial communities, we also included soil properties, i.e., nutrients and pH are covariates for the model. For soil nutrients, we first determined correlation among the variables using Pearson correlation test and included uncorrelated and weakly correlated factors, leading to total soil carbon and nitrogen (%), Zn, Cu, K, S, Na, and pH. The glm analysis was run with quassipoisson or log-linked Poisson distribution (for counts) where applicable. We conducted similar analyses with Shannon diversity as response variables separately. Where applicable, post hoc pairwise analyses of contaminant type was conducted using the emmeans package v 1.10.3 [30]. To further assess the effect sizes on the direction and magnitude of contaminant impacts of bacterial diversity relative to control, a log response ratio (LRR) test comparing responses in oiled or metal contamination relative to control was conducted. The LRR is the log of the ratio of two *ENS_PIE_* means, where the mean of either contaminant history (oiled or metal) is divided by a control mean (control site). We performed the analyses separately for oiled and metal environment using the LRRd function with bias correction set to default from the SingleCaseES v. 0.7.2 package [31]. 

To determine if shifts in bacterial ASV compositions (equivalent to beta-diversity) occurred according to contaminant types, we conducted a permutational multivariate analysis of variance (PERMANOVA) to partition sources of variations. We used the Bray–Curtis dissimilarity matrix, which accounts for abundance, as response variable, with contaminant type, the aforementioned soil nutrients and pH as predictor variables. The PERMANOVA model was run using the adonis2 function in the vegan package, with 999 permutations. To further visualize and confirm compositional shifts, we conducted distance-based redundancy analysis (db-RDA) on the same Bray–Curtis distance matrix using the forward selection model run at pstep = 500,000 to test for significant factors influencing shifts. 

### 2.4. Bacterial Functional Potential Analyses

To examine how exposure to contaminants affected the bacterial functional potential, we first determine the number of metabolic pathways identified, i.e., representative of the functional diversity potential by calculating the number of pathways (functional richness) and functional Shannon diversity index represented in the bacterial communities using similar approach described above (ASV diversity). We conducted similar glm analyses described above, but with the diversity metrics derived from the metabolic pathways as the response variables, i.e., functional richness and Shannon diversity. Next, we conducted similar PERMANOVA and db-RDA analyses on the potential functional differences among bacterial communities (i.e., Bray–Curtis dissimilarity matrix derived from the abundance-weighted metabolic pathway compositions; hereafter, functional differences) with the same model setup described above. To examine if and which metabolic pathways drive functional potential patterns according to contaminant history, we determined the pathways that are strongly associated with each environment by conducting a species indicator analysis. We used the multipatt function of the indicspecies package [32]), with the association function “r.g.” and max.order = 1 parameter settings and significance tested with 9999 permutations. The center-log ratio transformed data of predicted metabolic pathway abundance was used in the analyses.

Exposure to contaminants can differentially affect species diversity and functional potential of bacterial communities. To examine this, we first visualize overall patterns between ASV diversity and functional potential (richness) by plotting *ENS_PIE_* diversity against functional richness to determine the type of regression model to fit, i.e., linear or exponential regression. We then determined the significance of the relationship between species and functional diversity by fitting an exponential regression model on the ASV and functional richness potential across all samples (paired ASV and metabolic richness of each sample) using the model: log (functional richness) ~log (ASV *ENS_PIE_*). Next, we investigated whether and how exposure to contaminants shifts relationship between species diversity and functional richness potential, i.e., “decoupling” by conducting similar regression models but only within each respective environment, i.e., within oiled or metal or uncontaminated. 

### 2.5. Soil Property Analyses

We also analyzed whether soil nutrients and pH significantly varied across the three environments. To do so, we conducted separate analysis of variance (ANOVAs) for each nutrient and pH with contaminant type as the only predictor variable. 

All statistical analyses including ASV and functional potential were run in R, with all figures created using ggplot2 [33]. 

## 3. Results

### 3.1. Soil Property Analysis

Analyses of soil nutrients and pH showed differences across the three environments. Specifically, total soil %C, K, and Ca significantly differed among metal, oil, and uncontaminated environment while total N did not differ among the three sites (Table S2). Total %C (mean = 3.75 ± 0.97) and Zn (mean = 29.22 ± 38.81 mg/kg) were highest in the oiled environment while P was highest in the uncontaminated environment (mean = 9.02 ± 3.55 mg/kg) and significantly declined (Table S2). Copper was more than five times as high in the oiled environment (mean = 2.1 ± 2.64 mg/kg) compared to uncontaminated and heavy metal environments (mean = 0.2 ± 0.06 mg/kg, and 0.36 ± 0.22 mg/kg, respectively). Magnesium (Mg), S, Na, and Ca also showed significant differences according to the environment (Table S2). pH, on the other hand, was the same regardless of the environment. 

### 3.2. Summary Statistics of Microbial Sequences

**Figure 1:**
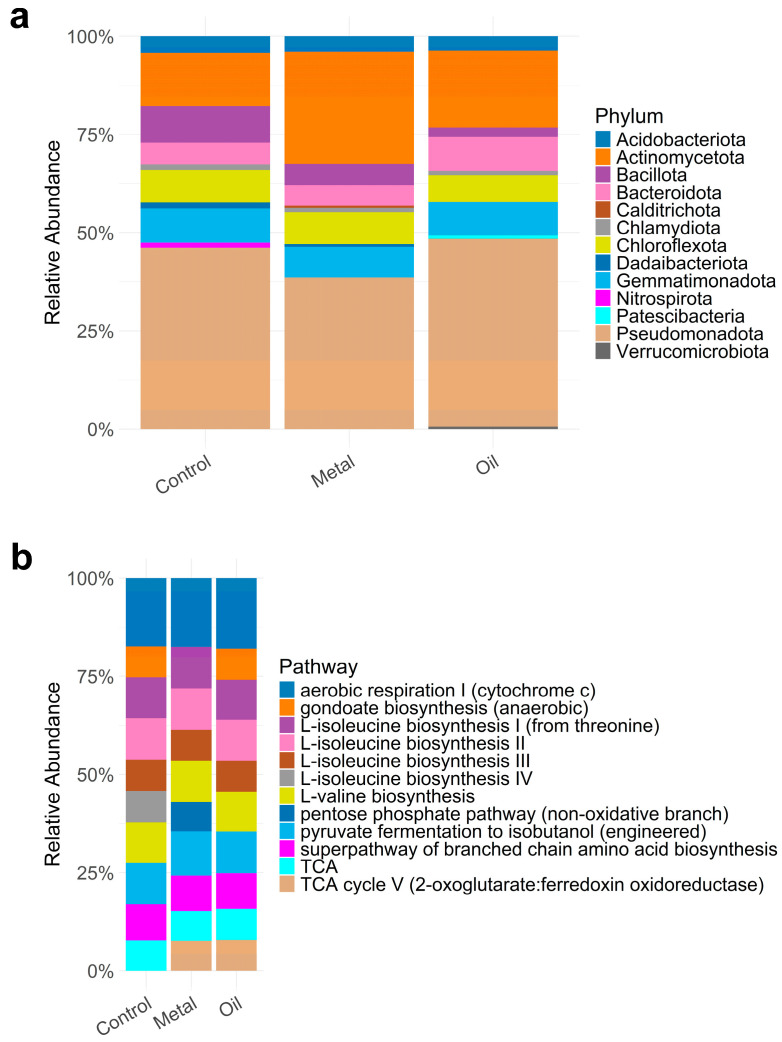
Top 10 (**a**) bacterial phyla and (**b**) predicted metabolic pathways identified across all soil bacterial samples.

We obtained ~1.5 million bacterial sequences across 45 soil samples, binned into 40,885 bacterial ASVs and further classified into 20 phyla. We only recovered 10 ASVs as archaea so these were excluded in the analyses. Species assignment of these ASVs across all samples indicate that *Pseudomonadota* (formerly, *Proteobacteria*) (42.82%) is the most dominant phylum, followed by *Actinomycetota* (formerly, *Actinobacteria*) (19.38%), *Gemmatimonadota* (8.04%), *Chloroflexota* (7.34%), and *Bacteroidota* (6.39%). The abundances of these phyla also differed across the three environments, with some phylum such as *Calditrichaeota* and *Verrucomicrobiota* abundant only in the metal and oiled environment, respectively (Figure 1a). Of these, *Alphaproteobacteria*, *Gammaproteobacteria,* and *Deltaproteobacteria* are the most dominant bacterial classes. PICRUSt2 analyses assigned predicted functional content (i.e., predicted metagenome content) to 458 metabolic pathways across all samples based on the high-throughput sequencing reads. The top abundant pathways were related to aerobic respiration I (cytochrome c), pyruvate fermentation to isobutanol (engineered), and biosynthesis (L-isoleucine biosynthesis I and II, L-valine biosynthesis) (Figure 1b).

### 3.3. Contaminant Impacts on Species Diversity of Bacterial Communities (H1)

**Figure 2:**
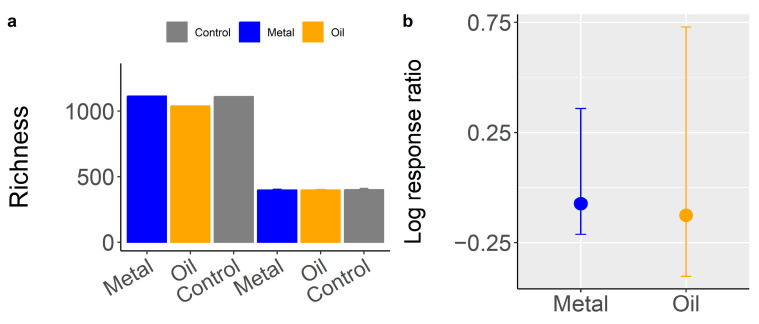
(**a**) Bacterial ASV *ENS_PIE_* and functional potential diversity according to contaminant type or history. (**b**) Log response ratio of bacterial ASV *ENS_PIE_* diversity in metal and oiled environment relative to bacterial communities in the control site. Error bars are confidence interval.

We first examined whether the ASV diversity of soil bacterial communities differed according to contaminant history. Generalized linear model analysis showed that *ENS_PIE_* (ASV alpha diversity) did not significantly differ among contaminant history, although overall bacterial *ENS_PIE_* diversity was lower in both oiled (mean = 192.465 ± 207.079) and metal (mean = 209.399 ± 95.925) environments compared to control or uncontaminated environment (mean = 226.1687 ± 71.377) (Figure 2a). Log response ratio analyses also showed no significant response to contamination, although *ENS_PIE_* diversity in oiled and metal-polluted environments exhibited reduced diversity levels (estimate = −0.126 and −0.073, respectively) relative to the uncontaminated or control site (Figure 2b). Similar results were obtained for Shannon diversity (Table 1). However, the presence of Cu (Estimate = 0.390, t = 2.658, *p* = 0.012) and Zn (Zn Estimate = −0.025, t = −2.088, *p* = 0.044) had significantly influenced bacterial *ENS_PIE_* diversity. Soil nutrients or pH did not alter bacterial Shannon or *ENS_PIE_* diversity (Table 1). Overall, these findings support our first hypothesis.

**Figure 3:**
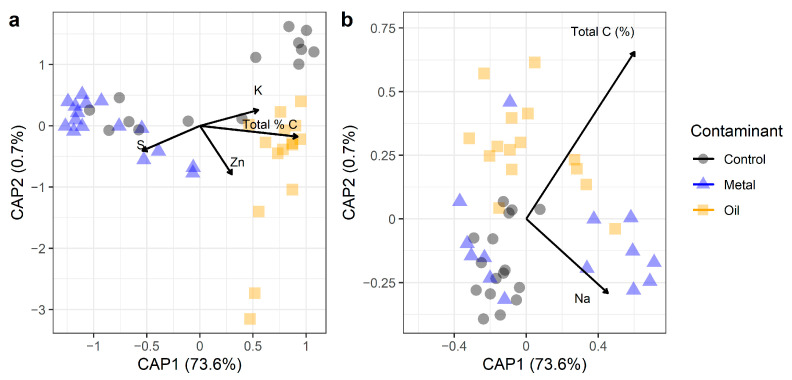
db-RDA of soil bacterial communities based on abundance-weighted Bray–Curtis dissimilarities in (**a**) ASVs and (**b**) predicted metabolic pathways, i.e., differences in functional potential.

PERMANOVA and db-RDA analyses based on Bray–Curtis dissimilarities among bacterial communities revealed that the greatest variations in bacterial community species composition can be attributed to contaminant history (R^2^ = 0.141, F = 3.842, *p* = 0.001) (Table 2, Figure 3a). Specifically, bacterial communities in the oiled environment were completely different from bacterial communities in the metal-polluted and control sites (Figure 3b). Total soil C (R^2^ = 0.050, F = 2.704, *p* = 0.001), Zn (R^2^ = 0.028, F = 1.498, *p* = 0.014), and Cu (R^2^ = 0.024, F = 1.314, *p* = 0.030) also had a weak but significant influence on shifts in bacterial community composition, along with sulfur and potassium (Table 2). There were no significant differences in bacterial community structure according to pH and total soil N. 

### 3.4. Contaminant Impacts of the Functional Potential of Bacterial Communities (H1)

The overall functional potential of bacterial communities significantly differed according to contaminant type based on the metabolic pathway composition but not on the functional richness (i.e., number of predicted metabolic pathways). Similar to ASV diversity, generalized linear analyses showed that contaminant type has no significant influence on the functional richness of soil bacterial communities (equivalent to functional alpha diversity), e.g., Control vs. Metal: z = −0.007, *p* = 0.995 (Figure 1b, Table 1). None of the soil nutrients or pH has a significant influence on the functional diversity (i.e., number of metabolic pathways and their relative abundances) of bacterial communities (Table 1). 

In contrast, PERMANOVA analyses indicate that total soil %C had the greatest influence (R^2^ = 0.184, F = 12.582, *p* = 0.001) on functional differences in metabolic pathways among bacterial communities. Contaminant type (R^2^ = 0.151, F = 5.177, *p* = 0.001) and sulfur (S) (R^2^ = 0.042, F = 2.891, *p* = 0.030) also had significant impacts on variations in functional potential among communities (Table 2). Further analysis based on db-RDA and visual representation indicate that bacterial communities also showed distinct functional compositions according to contaminant type (Figure 3b). For example, communities in the oiled environment clustered together, with some overlap with communities from metal-polluted and uncontaminated environments (Figure 3b). Similarly, communities in the metal and control sites exhibited clustering with some overlap from other environments. The first component explained most of the variations (CAP1, 47.9%), with total soil C and Na also explaining differences among bacterial communities (Figure 3b). Overall, these findings support our first hypothesis.

Indicator species analyses of metabolic pathways showed that different pathways significantly driving patterns in the functional potential of bacterial communities among the three environments. Twenty-five predicted metabolic pathways were strongly associated with metal environment including those related to sulfate reduction, while there were thirty-four pathways for the oiled environment and eighty-three pathways for the control environment. The top five indicator pathways for metal-contaminated environment were related to fatty acid metabolism (salvage, stat = 0.475, *p* = 0.002; and beta-oxidation, stat = 0.471, *p* = 0.003), sulfur reduction, and microbial degradation of various aromatic compounds (Table 3); whereas in the oiled sites indicator pathways were related to microbial degradation, e.g., aerobic, creatinine degradation, and glycine betaine degradation, which is implicated for osmoregulation [34]. For the control site, biosynthesis and methane oxidation were the top indicator pathways (Table 3). 

### 3.5. ASV Diversity vs. Functional Potential According to Contamination History (H2)

Regression analysis across all samples demonstrated a significant logistic relationship between ASV diversity and functional richness potential of bacterial communities (F_1,43_ = 10.24, *p* = 0.003), partly supporting our second hypothesis. However, analyses within each environment revealed that this relationship was only maintained under the oiled environment (F_1,13_ = 14.650, t = 3.828, *p* = 0.002). Species diversity and functional richness potential were "decoupled", i.e., not significantly correlated in both metal (F_1,12_ = 1.776, t = 1.333, *p* = 0.207) and uncontaminated or control (F_1,14_ = 1.028, t = 1.014, *p* = 0.328) environments. 

## 4. Discussion

Our study adds to the body of knowledge demonstrating the persisting impacts of environmental pollutions on soil microbial communities [2,35,36]. It also provides insights into how different types of small-scale contaminations alter both the species diversity and functional potential of soil bacterial communities. While bacterial ASV diversity and functional richness potential based on predicted metabolic pathways did not change in contaminated relative to control sites, bacterial communities showed distinct species composition and functions among oiled, metal, and uncontaminated environments. This indicates the strong influence of contaminants in shaping both the taxonomic and functional potential of bacterial communities. We also found different degrees of linkages between species diversity and functional richness depending on the environment. Overall, these findings suggest that the soil bacterial communities might recover in their diversity level years after initial exposure to small-scale contaminations; however, the resulting communities diverge in species composition and functional potential. Our results also highlight that exposure to contaminant, depending on the type, can lead to either strengthened linkages between species diversity and functional potential, as observed in oiled environment, or "decoupled" species diversity vs. functional potential as found in metal-polluted environment. 

Long-term exposure to oil and heavy metal contaminants in the gulf coastal prairies have led to soil bacterial communities demonstrating strongly distinct communities, although the (α) diversity levels are similar across the three environments. These shifts in ASV composition (β diversity) were also accompanied by changes in predicted metabolic functions across bacterial communities as they also exhibited clustering based on functional similarities. Our findings are in line with other studies where the impacts of chronic and/or long-term exposure to heavy metal contaminants were observed only in shifts in community composition and metabolic functions without affecting α- diversity [16,17,37]. For example, increase in heavy-metal resistance genes that were mostly from bacterial phyla *Pseudomonadota* and *Bacteroidota* had been observed in long-term metal-contaminated sites [16]. These differences could reflect the nature of the contaminants within the environment, with heavy metals and oil promoting the growth of different bacterial species depending on their tolerance to pollutants. For instance, heavy metals such as Pb, As, and Ba can promote bacterial taxa such as *Klebsiella* with high metal tolerance [35,38]. Different bacterial taxa also utilize various mechanisms for tolerating different heavy metals/metalloids. For instance, some members of *Betaproteobacteria* can oxidate and immobilize arsenic (As) via exopolysaccharide production while others belonging to the phylum *Bacillota* (formerly *Firmicutes*) oxidize/reduce metals [39,40]. Here, we also observed an increase in the abundance of *Actinomycetota* in the metal environment relative to oiled and control environments. Some members of this phylum such as *Micrococcus luteus* and *Tsukamurella paurometabola* are known to resist and bioadsorb Zn, Cu, and Pb [40]. 

Similarly, oil pollution can promote growth and survival of PAH degraders like Sphingomonads over time as some bacterial taxa can use different types of PAH as their sole carbon source [41]. Several members of *Actinomycetota,* e.g., *Mycobacterium vanbaalenii* have been shown to be highly adapted to the low bioavailable HMW substrates (k-strategists) due to the presence of multiple copies of different dioxygenase genes [42]. Bacterial species isolated from oil-contaminated environments such as *Rhodococcus qingshengii* has been demonstrated to harbor genes that can potentially degrade oil components including polychlorinated biphenyls [43]. Some species and strains of *Rhodococcus* are known to degrade oil [43,44,45], thus raising their possibility as bioremediation tool. Other studies on oil contamination also showed enrichment of *Gammaproteobacteria* [46] and *Pseudomonadales* [47], the latter causing shifts in indigenous microbial communities. In our study, the most abundant bacterium in the oiled environment belongs to the family Parvularculaceae (can only be identified up to family level), which is strictly aerobic and chemoheterotrophs. We also detected *Pelagibius, Desulfuromonas, Balneolaceae,* and *Rhodovulum* sp., which were all highly abundant only in the oiled environment. *Desulfuromonas* has been found to be enriched in a highly oiled environment in a controlled experiment [48]. Unfortunately, identification down to species level was not possible based on our taxonomic assignment. We note that the ecoregion in which our sites belong to (i.e., the gulf coast prairie/coastal dune barrier islands) is understudied; thus, it is possible some of the indigenous soil bacteria might not have been previously characterized elsewhere. Thus, it is possible that exposure to different contaminants led to divergent communities among oiled, metal, and control environments, without any changes in number of species. Certainly, previous studies demonstrated that oil contamination can lead to altered or shifted microbial communities compared to non-polluted sites [2,46].

The differences in functional potential of bacterial communities between metal and oiled environments are further corroborated by the varying metabolic pathways that are strongly associated with each environment. For instance, metabolic pathways related to the degradation of different carbon sources such as androstenedione, glucose, and creatinine are strongly associated with oiled environments. Oil or petroleum pollutants are often used as carbon and nitrogen sources of many bacterial taxa during biodegradation through aerobic and anaerobic metabolic pathways [14], thus explaining strong associations with biodegradation pathways. Meanwhile, bacterial communities in heavy metal environments showed a wide range of indicator pathways including various types of biosynthesis, sulfur oxidation and sulfate reduction, and a few aromatic degradations. As our study sites are spatially close to one another, these findings further indicate that contamination might lead to divergent soil bacterial communities in the gulf coast prairie dune/marsh habitats. 

Soil nutrients also demonstrated significant but weak effects on bacterial communities. Soil nutrients such as K, Na, and Ca are essential for bacterial growth and metabolism and thus can also influence community structure. While gulf coast prairie dunes and marshes are typically nutrient-poor environments, in this study, soil nutrients among the three sites significantly differed despite being in close proximity. Thus, it is also a possibility that the presence of contaminants in the soil could interact with bioavailable nutrients. For example, Zn was hundred-fold higher in the oiled environment compared to metal or control sites, and PERMANOVA analysis indicates Zn has a strong influence on bacterial species compositions. Unfortunately, we were unable to examine the degree to which their interactions might shape bacterial responses due to lack of statistical power. 

The strong linkages between bacterial ASV diversity and functional richness potential that we found in the oiled but not in metal or uncontaminated environment further highlight differential bacterial response to contaminations. These findings could imply that exposure to oil has resulted in bacterial communities that might not be functionally redundant (i.e., high number of different species within the various functional groups or guilds). On the other hand, bacterial communities exposed to heavy metals might have maintained their functional redundancy given that nearby control sites also showed potential redundancy. While it is generally considered that soil microbial communities exhibit redundancy in functions due to their high taxonomic diversity [7,12], some studies challenge this idea due to strong linkages between species and functional diversity, e.g., [49]. Interestingly, we observed a lack of significant correlation between species diversity and functional richness potential in the control or uncontaminated environment, indicating that perhaps this might be a natural phenomenon in the gulf coast prairie dunes and marshes. However, we interpret this with caution given that our study reflects only the functional potential of the bacterial communities, and we have not explicitly examined functional redundancies. 

We recognize that inferring microbial functions from 16S rRNA genes presents a challenge as it does not capture the active metabolic genes; rather, it reflects only the functional potential of these soil bacterial communities. For instance, actual microbial functional activities may vary while the abundances of predicted functional genes may be stable under specific environmental conditions. Thus, it is possible that the impacts of contaminants might be more muted when looking at functional potential of the whole community compared to the active members within the community. Further work is warranted to fully determine how environmental disturbances can decouple taxonomic diversity and functional diversity of active community members.

Our study has practical applications as soil bacteria are often used as indicators of soil quality and health and have been explored for their potential for remediation of contaminated soils [35]. Given the role of barrier islands in coastal protection and structural framework for many coastal and estuarine habitats, assessing the responses of soil bacterial communities to different contaminants is critical for future management, protection, and conservation of these habitats. 

## Figures and Tables

**Table 1:** Generalized linear model analyses results for bacterial ASV *ENS_PIE_*, ASV Shannon diversity and functional richness potential. The functional richness potential was based on the presence/absence of predicted metabolic pathways from the PICRUSt2.

Factors	ASV *ENS_PIE_*		ASV Shannon Diversity		Functional Richness Potential	
	Estimate	*p*-Value	Estimate	*p*-Value	Estimate	*p*-Value
Intercept	6.434	<0.001	1.964	0.000	6.071	<0.001
Control vs. Metal	0.255	0.726	0.023	0.836	0.000	1.000
Control vs. Oil	−0.366	0.748	0.002	0.999	−0.006	0.986
Metal vs. Oil	−0.111	0.963	0.022	0.829	−0.006	0.976
Total C (%)	−0.091	0.502	−0.018	0.203	0.000	0.980
Total N (%)	−3.470	0.671	0.344	0.633	0.190	0.708
Cu	0.390	0.012	0.026	0.135	0.001	0.955
Zn	−0.025	0.044	−0.001	0.266	0.000	0.915
K	−0.001	0.376	0.000	0.531	0.000	0.727
S	0.001	0.652	0.000	0.305	0.000	0.588
Na	0.000	0.828	0.000	0.521	0.000	0.980
pH	−0.094	0.576	−0.015	0.442	−0.011	0.403

**Table 2:** PERMANOVA analysis of bacterial ASV (top) and functional potential compositions based on abundance-weighted Bray–Curtis distances among soil bacterial communities. Df is degrees of freedom.

ASV Composition					
**Predictor**	**Df**	**Sum of Squares**	**R^2^**	**F**	***p* Values**
Contaminant	2	2.669	0.141	3.842	**0.001**
Total C (%)	1	0.939	0.050	2.704	**0.001**
Total N (%)	1	0.425	0.022	1.224	0.108
Zn	1	0.521	0.028	1.499	**0.014**
Cu	1	0.456	0.024	1.314	**0.030**
K	1	0.540	0.029	1.553	**0.025**
S	1	0.634	0.034	1.826	**0.003**
Na	1	0.385	0.020	1.107	0.232
pH	1	0.530	0.028	1.525	**0.034**
Residual	34	11.811	0.625		
Functional potential composition					
Contaminant	2	0.022	0.151	5.177	**0.001**
Total C (%)	1	0.026	0.184	12.582	**0.001**
Total N (%)	1	0.004	0.025	1.719	0.126
Zn	1	0.002	0.014	0.947	0.377
Cu	1	0.002	0.015	1.044	0.328
K	1	0.004	0.027	1.877	0.114
S	1	0.006	0.042	2.891	**0.030**
Na	1	0.004	0.026	1.758	0.126
pH	1	0.003	0.020	1.374	0.206
Residual	34	0.071	0.496		

Bold values indicate significant values.

**Table 3:** The top five metabolic pathways that are most strongly associated with each specific environment based on species indicator analysis. Bold numbers indicate significant *p*-value (<0.005).

Pathway	Stat	*p*-Value
**Heavy metal history**		
fatty acid beta-oxidation I (generic)	0.475	**0.0021**
fatty acid salvage	0.471	**0.0034**
4-aminobutanoate degradation V	0.445	**0.0043**
protocatechuate degradation II	0.420	**0.0089**
pyruvate fermentation to propanoate I	0.418	**0.0047**
**Control**		
hexitol fermentation to lactate, formate, ethanol and acetate	0.580	**0.0001**
L-methionine salvage cycle III	0.577	**0.0001**
S-methyl-5-thio-α-D-ribose 1-phosphate degradation I	0.577	**0.0001**
formaldehyde assimilation II (RuMP Cycle)	0.573	**0.0001**
formaldehyde oxidation I	0.569	**0.0001**
**Oil history**		
androstenedione degradation I (aerobic)	0.614	**0.0001**
glycine betaine degradation I	0.556	**0.0003**
beta-alanine biosynthesis II	0.538	**0.0001**
superpathway of hexuronide and hexuronate degradation	0.518	**0.0003**
creatinine degradation II	0.500	**0.0012**

## Data Availability

The original data presented in the study are openly available at NCBI SRA BioProject PRJNA1128733. R codes used in the statistical analyses are publicly available under Lumibao Lab Github (https://github.com/lumibaolab, accessed on 30 June 2024).

## Supplementary Materials

The following supporting information can be downloaded at: https://www.mdpi.com/article/10.3390/microorganisms12071460/s1, Table S1: TableS1_sample_info.pdf; Table S2: Mean values of soil nutrients (kg/mg).
