# Supplementary material for: Long-Term Contaminant Exposure Alters Functional Potential and Species Composition of Soil Bacterial Communities in Gulf Coast Prairies
Source: Microorganisms. 2024 Jul 18;12(7):1460. doi: 10.3390/microorganisms12071460 (PMC11279120; doi:10.3390/microorganisms12071460)
Supplement: Supplementary file 1 [file microorganisms-12-01460-s001.zip › Table S2.pdf]

Table S2. Mean values of soil nutrients

Soil properties	Metal	Oil	Control
Ca	8098.12 ± 3666.14[a,b]	13613.24 ± 5077.74[a,b]	1609.95 ± 1570.17
Cu	0.36 ± 0.22	2.1 ± 2.64[a,b]	0.2 ± 0.06
Mg	683.25 ± 226.08	292.47 ± 50.27[a,b]	671.9 ± 357.4
P	3.49 ± 1.91[a]	3.63 ± 5.91[a]	9.02 ± 3.55
K	338.3 ± 127.38[a]	258.83 ± 95.71[a]	157.51 ± 73.69
Na	3251.51 ± 2384.63	1242.69 ± 797.77[a,b]	4519.09 ± 2424.74
S	225.74 ± 189.62	123.05 ± 195.69[a]	307.92 ± 167.16
Zn	0.52 ± 0.32	29.22 ± 38.81[a,b]	1.24 ± 0.32
Total soil C (%)	1.63 ± 1.48[a,b]	3.75 ± 0.97[a,b]	0.16 ± 0.12
Total soil N (%)	0.05 ± 0.03	0.05 ± 0.01	0.04 ± 0.01
pH	8.02 ± 0.45	8.42 ± 0.52	8.07 ± 0.79

[a]significantly different from Control
[b]significantly different between oiled and metal environment
