# Supplementary material for: Long-Term Contaminant Exposure Alters Functional Potential and Species Composition of Soil Bacterial Communities in Gulf Coast Prairies
Source: Microorganisms. 2024 Jul 18;12(7):1460. doi: 10.3390/microorganisms12071460 (PMC11279120; doi:10.3390/microorganisms12071460)
Supplement: Supplementary file 1 [file microorganisms-12-01460-s001.zip › TableS1_sample_info.pdf]

sample_id	Contaminant	Lat	Long
Control.10	Control	27 40.533	97 11.068
Control.11	Control	27 40.523	97 11.055
Control.12	Control	27 40.501	97 11.051
Control.13	Control	27 40.548	97 11.043
Control.14	Control	27 40.332	97 11.009
Control.15	Control	27 40.324	97 10.591
Control.6	Control	27 40.556	97 11.065
Control.7	Control	27 40.558	97 11.056
Control.8	Control	27 40.553	97 11.054
Control.9	Control	27 40.542	97 11.044
CP1	Control	27 40.668	97 11.108
MP1	Metal	27 40.760	97 11.193
MP10	Metal	27 40.769	97 11.176
MP11	Metal	27 40.755	97 11.198
MP12	Metal	27 40.742	97 11.182
MP13	Metal	27 40.761	97 11.205
MP14	Metal	27 40.770	97 11.155
MP2	Metal	27 40.756	97 11.205
MP3	Metal	27 40.762	97 11.196
MP4	Metal	27 40.771	97 11.188
MP5	Metal	27 40.742	97 11.191
MP6	Metal	27 40.759	97 11.201
MP7	Metal	27 40.751	97 11.182
MP8	Metal	27 40.766	97 11.180
MP9	Metal	27 40.782	97 11.199
OT1	Oil	27 40.422	97 10.881
OT2	Oil	27 40.421	97 10.877
OT3	Oil	27 40.417	97 10.863
OT4	Oil	27 40.416	97 10.860
OT5	Oil	27 40.409	97 10.861
OT6	Oil	27 40.393	97 10.857
OT7	Oil	27 40.391	97 10.870
OT8	Oil	27 40.398	97 10.864
OT9	Oil	27 40.396	97 10.880
P1	Oil	27 40.17	97 10.892
P13	Control	27 40.462	97 10.976
P17	Control	27 40.469	97 10.980
P19	Control	27 40.473	97 10.980
P20	Control	27 40.472	97 10.982
P21	Control	27 40.474	97 10.983
P3	Oil	27 40.17	97 10.892
P5	Oil	27 40.17	97 10.895
P6	Oil	27 40.423	97 10.893
P7	Oil	27 40.422	97 10.891
P8	Oil	27 40.423[illegible]	97 10.887
